# Reward and Inhibitory Control as Mechanisms and Treatment Targets for Binge Eating Disorder

**DOI:** 10.1007/s11920-024-01534-z

**Published:** 2024-09-24

**Authors:** Ellen K. Pasquale, Allison M. Boyar, Kerri N. Boutelle

**Affiliations:** 1 San Diego State University/University of California San Diego Joint Doctoral Program in Clinical Psychology, 6363 Alvarado Court, Suite 103, San Diego, CA 92120 USA; 2 Department of Psychiatry, University of California San Diego, 9500 Gilman Drive, La Jolla, CA 92093 USA; 3 Herbert Wertheim School of Public Health and Human Longevity Science, University of California San Diego, 9500 Gilman Drive, La Jolla, CA 92093 USA; 4 Department of Pediatrics, University of California San Diego, 9500 Gilman Drive, La Jolla, CA 92093 USA

**Keywords:** Binge eating disorder, Reward, Inhibitory control, Impulsivity

## Abstract

**Purpose of Review:**

Recent research has highlighted alterations in reward and inhibitory control among individuals with binge eating disorder, identifying both constructs as potential targets for treatment. Treatments targeting reward and inhibitory control for binge eating disorder are emerging. This review aims to summarize the recent literature evaluating reward and inhibitory control in binge eating disorder compared to weight-matched controls using behavioral paradigms and neuroimaging. This review also aims to summarize recent literature evaluating treatments for binge eating targeting these mechanisms and highlights additional work needed in these areas.

**Recent Findings:**

Reward hypersensitivity and impaired inhibitory control are mechanisms underlying binge eating disorder. Individuals with binge eating disorder experience higher initial reward to food, and later, higher anticipatory reward but lower experienced food reward which maintains binge eating behavior. Treatments targeting reward and inhibitory control for binge eating include behavioral, computerized trainings, pharmacological, and neuromodulation treatments. The majority of trials are small but demonstrate promise in reducing binge eating and targeting theorized mechanisms. Larger, randomized trials are needed.

**Summary:**

Changes in reward and inhibitory control are present in individuals with binge eating disorder and treatments targeting these mechanisms demonstrate initial promise. Greater research is needed evaluating reward and inhibitory control simultaneously and with weight-matched comparison groups, as well as larger randomized trials that target both processes simultaneously.

## Introduction

Binge eating (BE) is associated with a myriad of consequences to physical health and mental health and contributes to poor quality of life [1–8]. BE is defined as the consumption of an objectively large amount of food with concomitant loss of control (LOC) over eating [9]. BE is a transdiagnostic component of multiple eating disorder (ED) diagnoses and is the defining characteristic binge eating disorder (BED) [9]. Individuals with BED are at elevated risk for developing chronic diseases due to the metabolic consequences of large volumes of highly palatable foods rapidly consumed during binge episodes [3, 10–12]. Cognitive behavioral therapy-enhanced for EDs (CBT-E) is the most evidence-based treatment for BED [13, 14]. While CBT-E demonstrates significant efficacy, meta-analyses suggest that only half of individuals with BED demonstrate BE abstinence after completing treatment [15, 16]. It is possible that the lack of durability of CBT-E is due to not sufficiently targeting underlying key mechanisms of BED.

A large and growing body of research suggests that abnormalities in reward and inhibitory control processes are associated with BE [17–19]. Greater research exists in understanding reward abnormalities in BED, which is heavily influenced by substance use research [20–23]. Genetic and neural vulnerabilities to food rewards may predispose BE behavior [24–26]. Over time, food consumption becomes associated with reward via both Pavlovian and operant conditioning [27, 28]. However, the receipt of the food reward does not increase with responding and may even decrease over time, necessitating the intake of even larger amounts of food to create the same reward response [29]. Thus, exaggerated signals of ‘wanting’ the food develop, despite lower experienced reward, which serves to maintain the BE behavior [29, 30]. While models of inhibitory control in BED are less developed, research suggests that reward abnormalities and deficits in inhibitory control processes exert influence on one another to promote BE behavior [20–22]. Both reward responsiveness and impaired inhibitory control are related to the dopamine system and share overlapping neural circuitry; thus, abnormalities in both areas may appear prior to the onset of the ED [20, 31, 32]. Models have been proposed to elucidate the relationship between reward and inhibitory control systems in BED and LOC eating in concert with other upstream constructs; however, these models have yet to be supported with data [26, 33, 34]. It is likely that diminished inhibitory control capacity cannot override the strong reward drive, leading to an imbalanced system. Data suggests that this imbalance between hyperactive reward responsivity and a hypoactive inhibitory control system may contribute to both the onset and maintenance of BED [35–37].

One of the challenges in evaluating reward and inhibitory control in individuals with BED is the overlap with obesity, as individuals with BED are estimated 3–6 times more likely to have obesity [38]. Data suggest that individuals with obesity without BED also have changes in reward and inhibitory control processes, independent of the presence of BE [39–41]. To understand the unique relationships between BE, reward, and inhibitory control independent of body weight, we focus on empirical studies that included weight-matched controls. Since there are already an abundance of review papers on these topics, we summarize review findings and more recent empirical studies are highlighted. Further, studies including only individuals with bulimia nervosa (BN) are not included, as the contribution of BE behavior on its own cannot be separated from the potential neurobiological contributions of purging or other compensatory behaviors.

The current review aims to provide a brief update of the literature (2018- early 2024), focusing primarily on samples of individuals with BED, to: 1) review behavioral studies of reward abnormalities associated with BED, 2) review behavioral studies of inhibitory control deficits associated with BED, 3) review neuroimaging studies of reward abnormalities and inhibitory control deficits in BED, where possible in conjunction with one another, 4) summarize recent research evaluating the efficacy of treatments targeting reward and inhibitory control processes, and 5) identify directions for future research. We utilize the Research Domain Criteria (RDoC) to define reward and inhibitory control [42–44].

## Reward Abnormalities and BED: Behavioral Assessments

A large body of research supports the presence of abnormalities in reward responsiveness among individuals with BED [45, 46]. Reward responsiveness refers to hedonic processes evoked by an impending or actual incentive [42]. RDoC categorizes reward responsiveness in 3 sub-constructs: 1) reward anticipation, 2) initial response to reward, and 3) reward satiation [42]. We focus on reward anticipation and initial response to reward as the majority of existing evidence falls in these domains.

Behavioral studies have primarily used eye tracking and reaction time paradigms to evaluate attention bias towards food cues as a proxy for reward, and/or electroencephalography (EEG) to evaluate event-related potentials (ERPs) to food and other (e.g., monetary) reward. One systematic review found that individuals who engage in BE in the absence of compensatory behaviors demonstrate increased attention bias towards food cues [47]. Two recent empirical studies have evaluated food reward with multi-modal behavioral approaches among individuals with BED. One study that combined EEG and eye tracking paradigms found that individuals with BED and overweight (n = 17) allocated more attention to food stimuli than non-food stimuli, and greater attention to food stimuli compared to a healthy weight control group (n = 17), but there were no significant differences compared to individuals with overweight without BED (n = 15) [48]. The second study evaluated ERPs relating to food and monetary reward among 40 individuals with BED and 40 age-, sex-, and BMI-matched controls without BED. The study also used ecological momentary assessment to assess BE in the BED group [49]. EEG data demonstrated that individuals with BED had both stronger anticipatory (contingent negative variation) and actual (reward positivity) neural reward activity in response to both food and monetary rewards compared to controls; however, greater frequency of BE during the ecological momentary assessment protocol was associated with stronger anticipatory but lower actual neural reward in response to food cues. These results suggest that increased BE frequency is associated with increased anticipatory reward but decreased experienced food reward, which supports previously described models of BED maintenance [49].

## Inhibitory Control Deficits and BED: Behavioral Assessments

A large body of evidence supports the presence of deficits in inhibitory control among individuals with BED and LOC eating [18, 40, 41, 50]. Inhibitory control specifically refers to the cognitive control that is involved in the inhibition and suppression of prepotent responses and motor responses [43, 44]. In addition to inhibitory control, we also describe research which uses the term ‘impulsivity,’ which is highly related to inhibitory control and is measured with similar paradigms [51].

Broadly, BED is associated with impaired inhibitory control in a number of review papers, though a recent systematic review demonstrated inconsistency in these relationships [50, 52, 53]. Studies have primarily used the Stop-Signal Task, Go/No-Go task, and a food-specific Go/No-Go task to evaluate inhibitory control among individuals with BED [54]. It is possible the mixed results in the identified systematic review were due to inconsistencies in the presence of weight-matched control groups or in adaptations of behavioral tasks used to measure inhibitory control [50].

One recent study used an eye tracking paradigm in conjunction with EEG to evaluate inhibitory control among individuals with overweight and BED (n = 24), individuals with overweight without BED (n = 23), and a healthy weight control group (n = 26) [55]. In this study, saccades and ERPs were evaluated during a food stimuli antisaccade task under negative mood induction. Individuals with BED demonstrated impaired inhibitory control measured via saccades compared to both control groups, and preliminary ERP evidence suggested less thorough conflict processing, which is related to impaired inhibitory control, in the BED and healthy weight samples. However, increased conflict processing latencies were observed in the sample with overweight without BED, which the authors hypothesized may serve as a compensatory strategy [55].

Functional near-infrared spectroscopy (fNIRS) is an ambulatory measurement of prefrontal cortex (PFC) activation collected via optical imaging that has been used to evaluate inhibitory control among individuals with BED in 2 recent studies, although both with small samples [56, 57]. The first evaluated PFC activation and relationships with impulsivity among individuals with obesity and BED (n = 13), obesity without BED (n = 15), and a healthy weight control group (n = 12) [56]. The study demonstrated overall PFC hypo-responsivity across tasks among both groups of individuals with obesity compared to the healthy weight control group. However, differences emerged contrary to expectation between individuals with obesity with and without BED, such that individuals with BED demonstrated greater right dorsolateral PFC response during the Go/No-Go task and greater right orbitofrontal cortex (OFC) response during a passive viewing task compared to the individuals with obesity only. This finding contrasts that of the second study, which used fNIRS to measure food-reappraisal in individuals with obesity and BED (n = 18) compared to individuals with obesity without BE (n = 14) [57]. fNIRS was used to evaluate PFC activation while participants viewed videos of food intake and were instructed to “resist” food stimuli. BE status was not related to differential activation of inhibitory PFC areas during the task. While fNIRS is established in neuropsychology, few studies have applied it to BE; thus, it is possible that task differences could contribute to the mixed results. Further, these mixed findings emphasize the need for and larger samples to determine which facets of inhibitory control may differentiate BED from the inhibitory control changes seen in obesity.

## Neuroimaging Studies of Reward and Inhibitory Control Changes in BED

The relationships between reward and inhibitory control circuits and BED have been reviewed in multiple recent review papers [17, 40, 58–62]. The reward system is composed of the midbrain/ventral tegmental area, the ventral striatum (including the nucleus accumbens; NAc), the PFC, and the OFC. The inhibitory control system includes the prefrontal regions, dorsal anterior cingulate cortex (dACC), inferior frontal gyrus, and presupplementary motor area (and subcortical regions including the subthalamic nucleus, dorsal caudate, ventrolateral and dorsolateral PFC [dlPFC], insula, and parietal cortex) [63]. Altered responses in the reward system coupled with diminished recruitment of prefrontal control circuitry are inversely related and believed to contribute to BE of palatable foods [32, 64].

Overall, data suggest that over time individuals with BED have less reward-mediated food consumption and more impulsive/compulsive food consumption, as evidenced by a shift from ventral-striatal to dorsal-striatal activity in response to food [59, 61, 65–67]. Individuals with BED show initial sensitization to food cues which is associated with increased activity in the ACC, insula, and OFC during the anticipation of food, and an increased striatal dopamine response in the caudate to receipt of food [18, 68]. Over time, this repeated release of dopamine in response to food cues is thought to downregulate presynaptic dopamine receptor activity, reduce presynaptic dopamine levels and dopamine release at rest [69, 70]. Review papers suggest that among individuals with BED, there are aberrant responses in both the reward and inhibitory control networks, including the insula, amygdala, middle frontal gyrus and occipital cortex, and increased regional cerebral blood flow and hypoactivity in the frontostriatal circuits in relation to food stimuli [61]. However, it is important to note that current research suggests that there is evidence for both hypodopaminergic and hyperdopaminergic activity among individuals with BED which may reflect the severity of BE, different stages in the disease process, or study differences and interpretations [69, 70].

Compared to reward, less is known about how the inhibitory control system relates to BED. A meta-analysis of studies found that neural activation during inhibitory control tasks among individuals with BED and obesity compared to weight-matched controls demonstrate a consistent pattern of reduced activation in the PFC (inferior frontal gyrus, ventromedial PFC, dorsolateral PFC) [41]. Another review showed that alterations in response inhibition system, including hypoactivity in the frontal regions and hyperactivity of the limbic regions, are more distinct when comparing individuals with obesity compared to healthy weight controls, and are aggravated in BED, although there are too few studies to understand differences between BED and weight-matched controls [62]. One recent study compared healthy weight individuals with BE (subthreshold BED) with weight-matched controls during behavioral inhibition tasks while undergoing fMRI. In this study, the participants with and without BE performed similarly on behavioral tasks but differed in PFC engagement. Specifically, the individuals with BE had lower activation of the right middle frontal gyrus and putamen during a Go/No-Go task, and higher activation of the left middle frontal gyrus during the Stop Signal Task [71]. Consistently, these results suggest that individuals with BED and subthreshold BED have hypoactivation in the frontal regions, particularly the PFC, which is considered a key region in self-regulation and allows for top-down inhibitory control.

Multiple studies suggest that there are disruptions in the functional architecture of reward and inhibition networks among individuals with BED when at rest. A systematic review reported that individuals with BE displayed a lower striatal dopamine release, a higher volume of and cerebral blood flow to cortical areas (ACC, insula and OFC) and the NAc, and lower frontostriatal connectivity [17]. Another study of individuals with BED compared to weight-matched controls showed hypoconnectivity between striatal regions which regulate reward processing, and prefrontal regions involved in cognitive and executive control, which was associated with increased binge frequency, suggesting that individuals with BED may be less able to regulate and inhibit responses to rewarding stimuli [45]. This hypothesis was supported by another study which showed that individuals with BED had weaker functional connectivity between the left lateral OFC and the right precuneus and the right dlPFC as compared to age-, sex-, and weight-matched controls [72]. Taken together, these studies suggest underlying architecture changes among individuals with BED may contribute to a diminished capacity to regulate reward-driven responses.

Recently, there has been emerging interest in evaluating BE in younger samples. There have been 3 cross-sectional papers comparing a sample of preadolescents (ages 9–10) with BED from the Adolescent Brain Cognitive Development (ABCD) study with weight-, gender- and age-matched controls. One study using voxel-based morphometry showed diffuse elevations in cortical gray matter density in those with BED, which spanned the prefrontal, parietal, and temporal regions, compared to controls [73]. Increased gray matter density can be indicative of reduced synaptic pruning or lower connectivity and is generally a hallmark of a lag in neural development. In a follow-up functional connectivity study, a seed-based approach was used to assess nodes in the reward (OFC, NAc, amygdala) and inhibitory control (dlPFC, ACC) networks. Results mirrored what is shown in adults, and found youth with BED compared to controls had reduced functional connectivity between the dlPFC and the amygdala, and between the ACC and OFC. [74]. In the most recent paper, task-evoked neural activity in the OFC, ACC, dlPFC and NAc in response to a monetary incentive delay task and the Stop Signal Task was compared between the youth with BED and controls. There were no significant differences between the youth with BED and the controls in this study [75]. A study among older adolescent girls evaluated a fMRI monetary reward task at age 16 and BE symptoms at baseline and 2 years later [76]. Data showed that greater ventromedial PFC and caudate response associated with winning money was correlated with greater severity of BE cross-sectionally but not longitudinally. In summary, studies among children and adolescents suggest that early-onset BED may be characterized by diffuse morphological abnormalities in gray matter density, and dysconnectivity between the reward and inhibitory control networks. Interestingly, differentiation in response to a monetary task was not seen in the younger children; however, it was found in the PFC and caudate in later adolescence, suggesting that there could be biological vulnerabilities to early BE which are expressed later in adolescence. Clearly, more research is needed to understand changes in these circuits over time in relation to BE and across development.

## Treatments Targeting Reward and Inhibitory Control Mechanisms to Reduce BE

While current evidence-based treatments for BED may target aspects of reward and inhibitory control, they do not target these constructs directly. The evidence highlighting reward changes and deficits in inhibitory control in BED can be leveraged to develop treatments directly targeting these underlying mechanisms with the goal of increasing efficacy and durability of results.

### Behavior Therapy

Several behavioral treatments, many of which are grounded in CBT, have been developed to address aspects of reward and inhibitory control directly in BED. Two randomized pilot trials have tested reward re-training, which uses principles from acceptance and commitment therapy, to reduce pleasure from palatable foods and increase pleasure from daily activities among individuals with transdiagnostic BE [77, 78]. In an initial trial comparing reward re-training to a waitlist control, reward re-training demonstrated large impacts on both hypo- and hyper-reward response via both self-report and fMRI, and demonstrated reductions in self-reported ED pathology [77]. However, in a second trial comparing reward re-training to supportive therapy, no significant differences in hypothesized mechanisms (food reward, day-to-day activity reward, or social support) were observed between groups; however, mediation analyses revealed at mid-treatment a significant indirect effect of reward re-training on lower global Eating Disorder Examination scores by way of decreased food reward (Power of Food Scale) [78]. One randomized trial piloted CBT enhanced with skills targeting inhibitory control for individuals with BED [79]. While the study did not result in significant differences in BE at post-treatment or follow-up, it did result in greater reductions in ED pathology at post-treatment in the inhibitory control group compared to a waitlist control [79]. A secondary analysis of fMRI data showed individuals who received the inhibitory control treatment demonstrated increased right PFC activity during response inhibition, which was correlated with decreased trait impulsivity after treatment [80].

Cue-exposure treatment for food (CET-F) has a more established evidence-base, and theoretically aims to reduce reward of palatable food through habituation during exposure and increase inhibitory control through learning to tolerate cravings and revising predictor errors [81]. A systematic review of 18 studies found CET-F reduced BE in both the short- and long-term in individuals with BED [81]. The Regulation of Cues (ROC) treatment incorporates CET-F with appetite awareness training, and showed significant reductions in BE over treatment and follow-up in a large randomized trial among individuals seeking treatment for BE, overeating, and weight management [82]. In a second recently completed trial evaluating ROC among Veterans with BE, initial results indicate that ROC outperformed CBT in reducing BE (manuscript under review). [83] Despite primarily small sample sizes, these studies provide evidence for the potential efficacy of behavioral treatments targeting reward and inhibitory control for BE, with the largest evidence base for CET-F.

### Computerized Trainings

Computerized trainings have been tested targeting the modification of attentional biases towards food (proxy for reward) and improving inhibitory control to reduce BE. Attention/approach bias modification (ABM) programs aim to train avoidance behavior to food cues to increase food-specific inhibitory control, and have been tested in 2 recent randomized trials, one among individuals with BED and BN (n = 56) and another among individuals with overweight/obesity with and without BED (n = 45) [84, 85]. While both studies favored ABM in reducing ED pathology, neither study demonstrated reductions in BE, albeit both had small samples. Computerized inhibitory control trainings (ICTs) for BE use repeated behavioral assessments such as the food-specific Go/No-Go task to train inhibitory control. One single-arm pilot study evaluated a virtual reality ICT which aimed to increase behavioral inhibition of automatic approach responses to palatable foods among individuals with 1+ weekly binge episodes. This study demonstrated large decreases in LOC eating at post-treatment and follow-up [86]. More research with larger randomized trials is necessary to fully evaluate the efficacy of computerized trainings targeting reward and inhibitory control for BE.

### Pharmacological Treatments

Pharmacological treatments that theoretically target reward and/or inhibitory control circuitry have been tested among individuals with BED, some of which were originally developed for type 2 diabetes or weight management but have been applied to BED. One potentially promising recent advancement is the application of GLP-1 agonists to BED; however, evidence for their efficacy in this population is still emerging [87–89]. GLP-1 agonists directly act upon GLP-1 receptors located primarily in reward-related brain regions and reduce brain responses to food pictures among individuals with type 2 diabetes and obesity [90, 91]. One retrospective cohort study demonstrated greater reductions in BE among individuals with BED prescribed semaglutide (GLP-1 agonist) compared to individuals prescribed lisdexamfetamine, topiramate, or a combination of semaglutide with lisdexamphetamine or topiramate [92]. To our knowledge, only one published RCT has evaluated a GLP-1 agonist in BED, which compared liraglutide to placebo. While the liraglutide group demonstrated higher BED remission, it was not significantly different from placebo and a pharmacy dispensing error significantly limited results [93]. It is important to note the potential for misuse and iatrogenic effects of GLP-1 agonists in clinical presentations of BE with significant dietary restriction such as BN [94]. Naltrexone/bupropion, which targets food reward by blocking opioid receptors, has demonstrated mixed results on reducing BE, but has demonstrated utility in the maintenance of BED remission after successful acute treatment [95–98]. Stimulants including lisdexamfetamine and methylphenidate, which target increasing inhibitory control processes by increasing PFC activity, have also been applied successfully to BED, with methylphenidate recently demonstrating effects comparable to CBT [99, 100]. A systematic review found consistent reductions in BE symptoms in BED with lisdexamfetamine, which is the sole currently FDA-approved pharmacological treatment of BED [101].

### Neuromodulation Treatments

There has been a recent increase in neuromodulation treatments for BED, primarily targeting inhibitory control mechanisms, albeit in mostly small pilot trials. These include transcranial direct-current stimulation (tDCS), transcranial magnetic stimulation (TMS), and neurofeedback via EEG and interventional fNIRS. Many review papers have been published, summarizing trials evaluating tDCS, TMS, and EEG-neurofeedback among individuals with BED and with BE and obesity [102–105]. More specifically, evidence for TMS in reducing BE is mixed [104, 105]. Evidence is somewhat stronger supporting tDCS among individuals with BED and EEG-neurofeedback combined with cue exposure in decreasing BE and food craving [104–106]. One small randomized trial applied interventional fNIRS with neurofeedback to BED, which demonstrated reduced LOC eating after fNIRS neurofeedback sessions among individuals with BED [107, 108]. These preliminary studies show promise, but the literature is nascent for these modalities, and larger trials are needed to fully understand treatment mechanisms, the parameters of greatest effect, and if these modalities are best leveraged as stand-alone or adjunctive treatments.

## Directions for Future Research

There is sufficient evidence to suggest that alterations in reward and inhibitory control processes are associated with BED, although our understanding of the nuances, mechanisms, and their relationships is still emerging. Thus, there is a need for more studies that measure both reward and inhibitory control simultaneously to better understand the imbalance that occurs in BED. In neuroimaging research, greater studies evaluating functional connectivity between the reward and inhibitory control systems may better elucidate this imbalance. Further, it is still a challenge to identify differences specific to BED that are not accounted for by changes that may be attributable to obesity. Larger, high-quality studies with weight-matched control groups will help elucidate these differences.

Longitudinal studies are needed to evaluate the temporality of the relationship between changes in reward, and inhibitory control and how these changes contribute to the development and maintenance of BED. Specifically, differing findings relating to the directionality of reward changes in BED have been observed, with some studies reporting evidence for a hyperactive reward system and others reporting evidence for a hypoactive reward system. Longitudinal studies may better elucidate how reward response may change over time in BED to clarify this difference. Finally, many treatments for BED that target reward and inhibitory control mechanisms show promise by targeting these upstream constructs directly. Larger trials are needed, specifically RCTs to help solidify the state of the evidence for these modalities. Since both reward and inhibitory control systems are implicated, treatments that target both systems simultaneously may be the most effective and may lead to the most durable results.

## Conclusions

In conclusion, a large body of evidence consistently supports cross-sectional associations between changes in reward and inhibitory control deficits with BE, and emerging evidence supports the presence of longitudinal associations. Recent studies suggest an imbalance between either a hyper- or hypo-active reward system and hypoactive inhibitory control system contribute to the development and maintenance of BED pathology. While treatments targeting reward and inhibitory control are nascent, they show promise. More research is needed evaluating both reward and inhibitory control simultaneously to better understand the potential imbalance between the processes contributing to BED pathology, and treatments that target both processes simultaneously may demonstrate the strongest results.

## Data Availability

No datasets were generated or analyzed during the current study.
